# Biological Interaction and Imaging of Ultrasmall Gold Nanoparticles

**DOI:** 10.1007/s40820-023-01266-4

**Published:** 2023-12-04

**Authors:** Dongmiao Sang, Xiaoxi Luo, Jinbin Liu

**Affiliations:** Key Laboratory of Functional Molecular Engineering of Guangdong Province, School of Chemistry and Chemical Engineering, South China University of Technology, Guangzhou, 510640 People’s Republic of China

**Keywords:** Ultrasmall gold nanoparticle, Cellular interaction, Organ interaction, Tumor interaction, Bioimaging

## Abstract

- The ultrasmall gold nanoparticles (AuNPs), serving as a bridge between small molecules and traditional inorganic nanoparticles, create significant opportunities to address many challenges in the health field.
- This review discusses the recent advances in the biological interactions and imaging of ultrasmall AuNPs.
- The challenges and the future development directions of the ultrasmall AuNPs are presented.

**Abstract:**

Ultrasmall gold nanoparticles (AuNPs) typically includes atomically precise gold nanoclusters (AuNCs) and AuNPs with a core size below 3 nm. Serving as a bridge between small molecules and traditional inorganic nanoparticles, the ultrasmall AuNPs show the unique advantages of both small molecules (e.g., rapid distribution, renal clearance, low non-specific organ accumulation) and nanoparticles (e.g., long blood circulation and enhanced permeability and retention effect). The emergence of ultrasmall AuNPs creates significant opportunities to address many challenges in the health field including disease diagnosis, monitoring and treatment. Since the nano–bio interaction dictates the overall biological applications of the ultrasmall AuNPs, this review elucidates the recent advances in the biological interactions and imaging of ultrasmall AuNPs. We begin with the introduction of the factors that influence the cellular interactions of ultrasmall AuNPs. We then discuss the organ interactions, especially focus on the interactions of the liver and kidneys. We further present the recent advances in the tumor interactions of ultrasmall AuNPs. In addition, the imaging performance of the ultrasmall AuNPs is summarized and discussed. Finally, we summarize this review and provide some perspective on the future research direction of the ultrasmall AuNPs, aiming to accelerate their clinical translation.
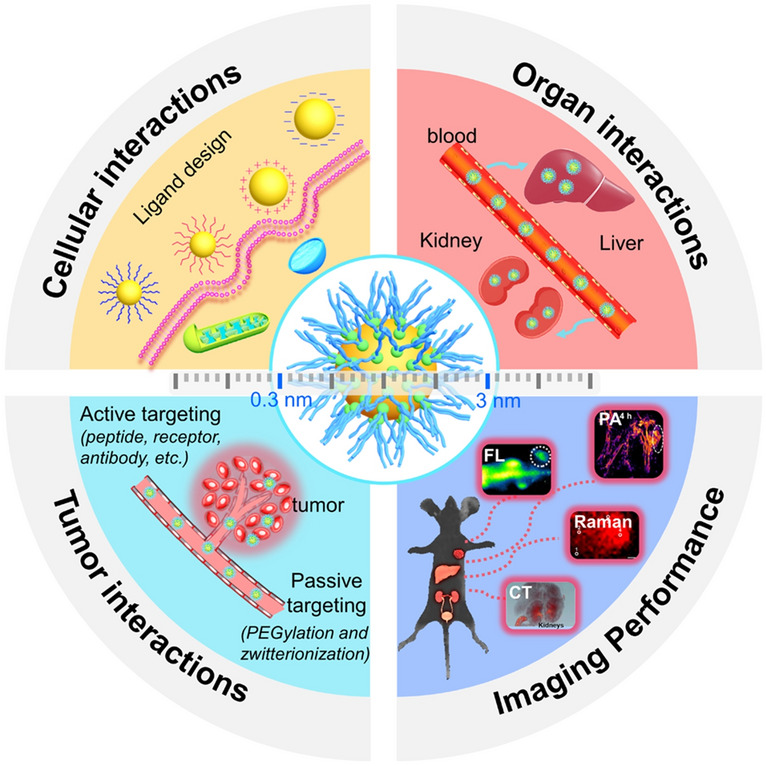

## Introduction

Ultrasmall gold nanoparticles (AuNPs) consist of atomically precise gold nanoclusters (AuNCs) or AuNPs with a core size below 3 nm [1–4], which possess a unique core–shell structure with sizes between small molecules and conventional nanoparticles [5, 6]. As a result of the sharp size shrinking, the continuous energy levels of AuNPs become discrete [7, 8], resulting in distinct optical and electronic properties. After systematical regulation of both the core and ligand layers of the ultrasmall AuNPs, the intrinsic excitation and emission wavelengths of AuNPs can be facilely tuned from visible region to the second near–infrared window (NIR-II, 1000–1700 nm) [9, 10], facilitating the bioimaging investigation at both cellular and in vivo levels. Besides, the ultrasmall size endows the AuNPs with the characteristics of small molecules, while maintain the properties of inorganic nanoparticles with modifiable ligand layers towards multifunctional chemical modification [11, 12]. The quantum confinement effect resulting from the ultrasmall size also allows ultrasmall AuNPs to undergo electron transitions under laser excitation, enabling the possibility of optical imaging and photodynamic therapy [13–15]. Furthermore, the high atomic number of Au (Z = 79) provides high X-ray absorption coefficient, making ultrasmall AuNPs suitable for computed tomography (CT) imaging and radiation sensitization, thus possessing both imaging and therapeutic capabilities [16–19].

Currently, various large-sized plasmonic AuNPs [20–23] and small molecules (e.g., dyes and drug molecules) [24–26] have been developed for the biological imaging, diagnostics, and therapeutics. Many excellent review articles have summarized the factors influencing the biological interaction and imaging performances of the plasmonic AuNPs and small molecules [27–32]. However, a comprehensive review specifically focused on the biological interactions of the ultrasmall AuNPs is scarce. Serving as a bridge between small molecules and plasmonic AuNPs, the ultrasmall AuNPs possess the advantages of both small molecules and nanoparticles. Inside the biological system, ultrasmall AuNPs can rapidly distribute throughout the body like small molecules, undergo rapid renal clearance, exhibit low non-specific organ accumulation, and avoid long-term toxicity [33, 34]. On the other hand, the ultrasmall AuNPs can also exhibit long circulation in the bloodstream, similar to traditional inorganic nanoparticles, and accumulate at tumor or diseased sites through the enhanced permeability and retention (EPR) effect [35, 36]. The emergence of ultrasmall AuNPs creates significant opportunities for the application-driven surface engineering strategies of ultrasmall AuNPs to regulate their biological behaviors and bioimaging performance towards future clinic translation [37, 38].

The ultrasmall AuNPs are formed from the aggregates of small amount of gold atoms protected by surface ligands (e.g., thiolate [39, 40], phosphine [41, 42] and alkynyl [43, 44]). In the past two decades, the synthesis of ultrasmall AuNPs have been rapidly developed [45–47], which can be categorized into two main synthetic routes: (i) gold salt (complex) reduction, and (ii) etching from plasmonic AuNPs. With the advances in the fundamental understanding of the underlying reaction process in size control (e.g., kinetic control and thermodynamic selection) [48, 49] and structural determination (e.g., X-ray crystallography and mass spectrometer) [50–54], the controlled synthesis of ultrasmall AuNPs have been widely reported, which showed not only enriched sizes, compositions, and structures but also attractive optical and biological properties for various advanced biomedical applications [55–57]. Since many excellent reviews have summarized the recent progress in the facile and robust synthesis of ultrasmall AuNPs with high reproducibility [58–64], we will not focus on the synthetic strategies in this review.

**Fig. 1:**
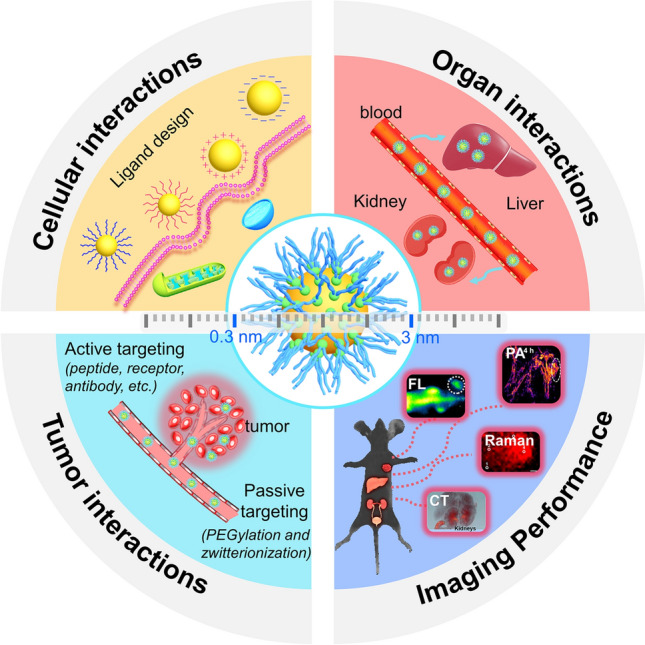
Summary of the biological interaction and imaging of ultrasmall AuNPs in this review Imaging Performance: fluorescence imaging [65]. (Copyright (2020), John Wiley and Sons), photoacoustic imaging [66]. (Copyright (2023), Royal Society of Chemistry), Raman imaging [67]. (Copyright (2023), American Chemical Society), CT Imaging [68]. (Copyright (2015), American Chemical Society)

In this review, we summarize the recent progress of ultrasmall AuNPs in biological interactions and the related imaging performance (Fig. 1). We firstly discuss the cellular interaction of ultrasmall AuNPs at the cellular level. Furthermore, we discuss the organ interactions of ultrasmall AuNPs, especially emphasized on the kidney and liver interactions. We also present the recent strategies for passive and active tumor targeting of ultrasmall AuNPs. Additionally, we elucidate the imaging performance of ultrasmall AuNPs including fluorescence imaging, CT imaging and other imaging modes. Finally, we critically comment on the biological interactions of ultrasmall AuNPs and present future perspectives to accelerate their clinical applications in address many challenges in the health field including disease diagnosis, monitoring, and treatment. By summarizing the factors that influence the biological interactions and imaging performance of ultrasmall AuNPs, we believe that with a better understanding of the in vivo interactions of the ultrasmall AuNPs will provide insight into the in vivo imaging and treatment, and ultimately will achieve clinical translation.

## Cellular Interactions

Nanoparticles play important roles in mediating the biological interactions, which requires a comprehensive understanding of the nano–bio interactions [69, 70]. Therefore, it is of great significance to investigate cellular uptake and trafficking mechanisms between nanoparticles and cells. The cellular interactions initially occur at the interface between cell membranes and nanoparticles, and would substantially affect the subsequent subcellular interactions and outcomes at the cellular level [71, 72]. Cellular uptake and intracellular transport highly depend on the surface physical and chemical properties of the ultrasmall AuNPs [73]. The fundamental understanding of the factors that affect cellular uptake, organelle distribution, and cytotoxicity will provide a foundation for further elucidations of the nano–bio interactions [72, 74, 75]. In this review, the factors including surface charge, surface coverage, hydrophobicity, surface functionality and concentration effect, will be summarized [76–81].

### Surface Charge Effect

**Fig. 2:**
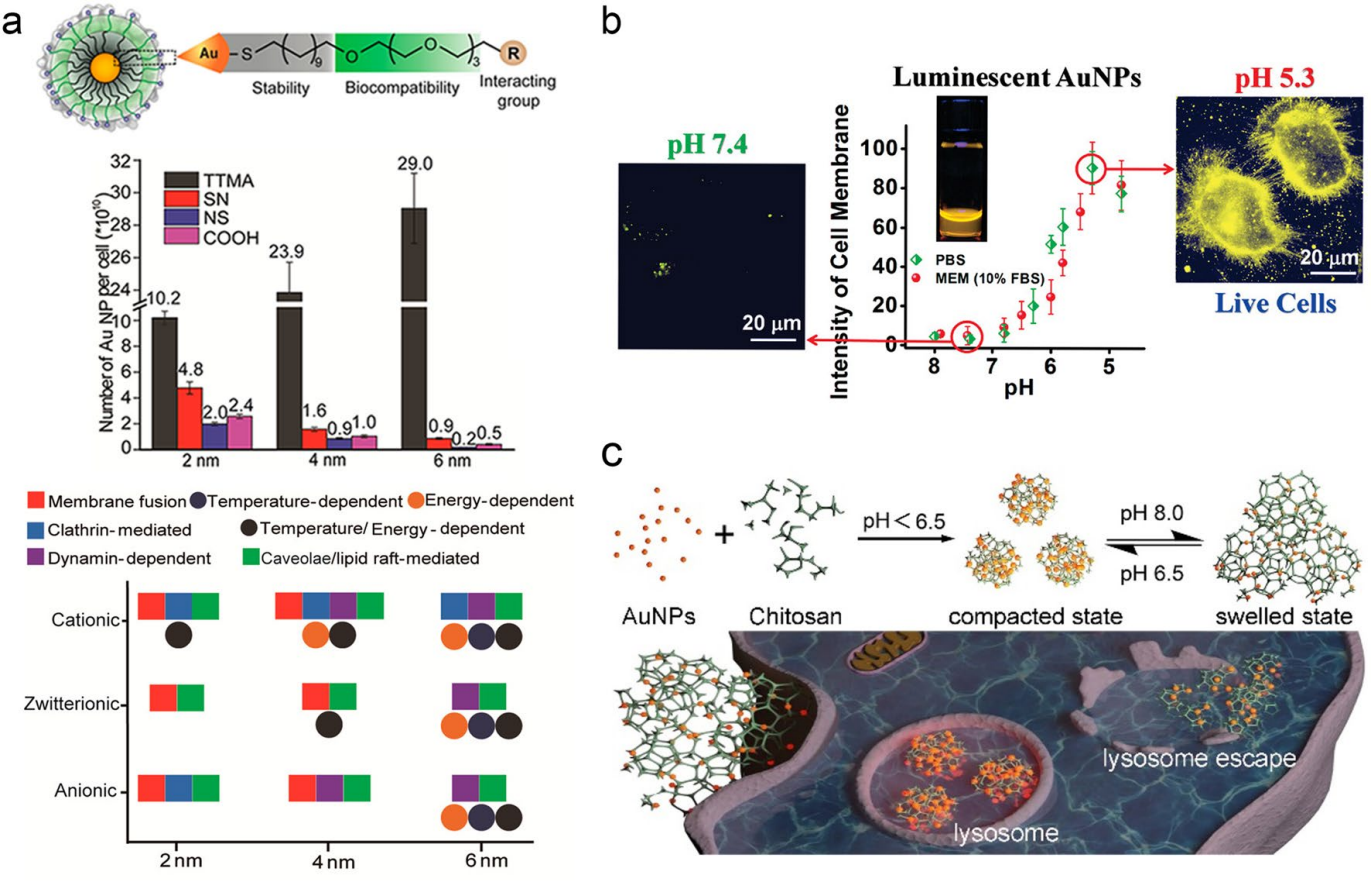
The effect of surface charge on cellular interactions of ultrasmall AuNPs. **a** Interplay of size and surface functionality on the cellular uptake pathway of ultrasmall AuNPs [86]. Copyright (2015), American Chemical Society. **b** Ultrasmall AuNPs with pH–dependent membrane adsorption [87]. Copyright (2011), American Chemical Society. **c** Schematic illustration of the endocytosis and lysosome escape of AuNPs@CS [88]. Copyright (2019), American Chemical Society

Electrostatic attraction plays important role in governing the cellular interactions of ultrasmall AuNPs, resulting from the negatively-charged phospholipids on the cell membrane [82–84]. Therefore, surface charge significantly affects cellular interactions of ultrasmall AuNPs. Typically, large-sized positively-charged AuNPs (e.g., 17.7 ± 1.6 nm) exhibited the much higher cellular uptake efficiencies than those of the neutral or negatively-charged ones, The uptake efficiency of positively-charged AuNPs was 5–10 times higher than that of neutral or negatively-charged AuNPs within 24 h [85]. In the investigation of the size-dependent cellular uptake of AuNPs, Rotello et al. investigated the effect of surface charge including positive, neutral, and negative on the cellular interactions of AuNPs with the core sizes of 2, 4 and 6 nm, respectively (Fig. 2a) [86]. The results demonstrated that the positively-charged AuNPs exhibited significantly higher cellular uptake efficiencies than those of the neutral or negatively-charged ones. However, the cellular uptake efficiencies of positively-charged AuNPs increased with the size increased from 2 to 6 nm, significantly different from those of the neutral or negatively-charged ones with decreased cellular uptake efficiencies. In addition, the internalization mechanisms were also different: the amphiphilic neutral AuNPs with size of 2 and 4 nm were mainly internalized through a membrane fusion mechanism, while the large-sized ones (6 nm) involved a clathrin-mediated endocytosis/lipid raft pathways, indicating that the internalization mechanisms undergo a size-dependent transition within the small size range. Therefore, the positively-charged ultrasmall AuNPs showed a significantly high cellular uptake, consistent with the plasmonic large ones, however, the interaction mechanisms require more systematic investigation.

Additionally, ultrasmall AuNPs with different surface charges can lead to different toxicities. Hussain et al. synthesized ultrasmall AuNPs (~ 1.5 nm) with different charges to investigate the relationship between surface charge and cellular toxicity [89]. The half maximal inhibitory concentration (IC50) value of both positively and negatively-charged AuNPs were less than 10 μg mL^−1^, while the neutral ultrasmall AuNPs had an IC50 value of 25 μg mL^−1^. All the ultrasmall AuNPs showed dose-dependent toxicity but with different mechanisms. Both the positively-and negatively-charged AuNPs induced cell death through apoptosis, whereas the neutral AuNPs caused cell necrosis. To effectively utilize the strong membrane binding capability of amino-functionalized ultrasmall AuNPs (AuNPs–NH_2_, 2.5 ± 0.4 nm) as well as reduce toxicity, Kim et al. proposed a host–guest system that utilized supramolecular chemistry for intracellular activation [90]. Threading of cucurbit[7]uril (CB[7]) on the surface of AuNPs-NH_2_ reduced the cytotoxicity via endosome sequestration, which increased the IC50 from 1.3 to 50 mM. The therapeutic effect was then induced through the introduction of 1-adamantylamine (ADA) to release AuNPs-NH_2_, resulting in in situ cytotoxicity of AuNPs-NH_2_.

pH regulates many cellular interaction processes and is considered as an indicator of disease progression [91, 92]. The strategies of pH-stimulated charge change of ultrasmall AuNPs were also developed. Zheng et al. synthesized ultrasmall AuNPs with pH-dependent membrane adsorption using the surface ligands of both GSH and cationic cysteamine (GC-AuNPs, Fig. 2b) [87]. The introduction of cysteamine ligand on the surface of GC-AuNPs induced the significantly decrease in negative charges with zeta potential values increased from − 29.8 ± 1.8 mV at pH 7.4 to − 15.7 ± 1.7 mV at pH 5.3, which caused significant enhancement in membrane adsorption at low pH values (e.g., pH 5.3). Rotello et al. synthesized pH–responsive ultrasmall AuNPs (2 nm) using alkoxyphenyl acylsulfonamide amide ligands [93]. The pH-responsive AuNPs exhibited selective cellular uptake and cytotoxicity. When the pH decreased from 7.4 to 6.0, the neutral AuNPs became positively charged, resulting in both increased toxicity and cellular uptake (~ fourfold). The pH-responsive ultrasmall AuNPs demonstrate the potential for selective treatment at tumor sites. In our group, using a conventional cationic polymer chitosan (CS, isoelectric point at 6.5) as a template [88], we constructed a pH-responsive self-assembled AuNPs (AuNPs@CS) with reversibly pH-dependent swelling and compacting structures at physiological pH range (pH 6.5–7.4), which showed pH-responsive cellular interaction capability and sensitive emission response toward subcellular location (Fig. 2c). At low pH values (e.g., pH < 6.5), the AuNPs@CS assembled into dense nanostructures (~ 23.5 nm) with fluorescence intensity increase, while at high pH values (e.g., pH 7.4), AuNPs@CS transformed into weakly emitting swelled structures and possessed the ability to escape from lysosomes. This pH-responsive self-assembled AuNPs@CS can be utilized for enhanced cellular uptake and intracellular optical tracking.

### Surface Coverage Effect

**Fig. 3:**
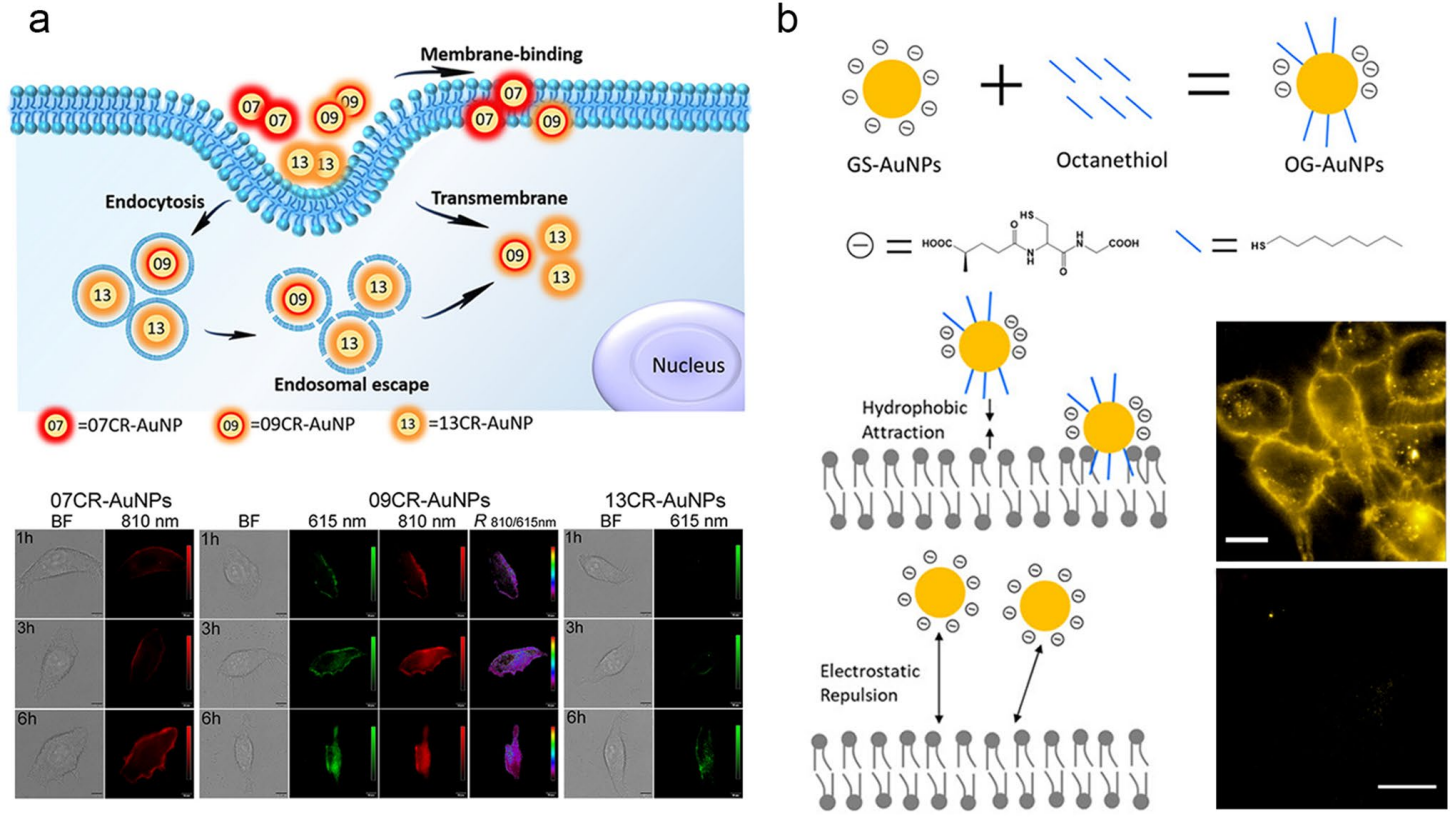
Effects of the surface coverage and hydrophobicity on the cellular interactions of ultrasmall AuNPs. **a** Surface coverage-regulated cellular interaction of ultrasmall AuNPs [94]. Copyright (2019), American Chemical Society. **b** Effect of hydrophobicity on nano–bio interactions of zwitterionic ultrasmall AuNPs [102]. Copyright (2018), American Chemical Society

The surface chemistry of ultrasmall AuNPs plays key roles in the establishment of interfaces between the metal core and cell surface. The surface coverage of AuNPs provides versatile toolboxes to modulate the cellular interactions of ultrasmall AuNPs. The cellular interactions of ultrasmall AuNPs (~ 2.0 nm) with different surface coverages (e.g., 29%, 32%, and 47%) were investigated in our group (Fig. 3a) [94]. The results showed that lower surface coverage (e.g., 29%) resulted in high membrane binding percentage (~ 82%, at 6 h incubation) with low cellular uptake, whereas high surface coverage (e.g., 47%) led to low membrane binding percentage (~ 36%) with high cellular uptake. In addition, the AuNPs with low surface coverage showed faster cellular interaction than those of the ones with high surface coverage. For larger-sized nanoparticles, ligand density mediates the conformational fluctuations of ligands, protein absorption repulsion, and cell adhesion strength [95, 96], all of which can regulate cellular interactions of larger-sized nanoparticles. Burda et al. investigated the effects of ligand density and molecular weight on large-sized AuNPs on cellular uptake and toxicity [97]. The AuNPs with the same core size (6 ± 2.5 nm) was prepared by adjusting the ratio of PEG:AuNPs ratios (100–300:1, PEG:AuNPs ratios) and molecular weight (0.55, 1, 2, and 5 kDa). With the increase of PEG:AuNPs (2000 Da) ratio (from 200:1 to 800:1), the viability of HeLa cells incubated with PEGylated AuNPs increased. When the proportion of PEGylated AuNPs synthesis reached the critical stability ratio, the cell viability reached the best. Moreover, cellular uptake decreased with the increase of PEG molecular weight. Therefore, by adjusting the surface ligand density of AuNPs, the physical and physiological properties can be adjusted and optimized for biomedical applications. At the ultrasmall size range, nanoparticles possess a larger surface area, amplifying the role of surface ligands, and the influence of ligand density on the cellular interactions of ultrasmall nanoparticles cannot be ignored. The modulation of surface coverage in ultrasmall AuNPs can be utilized to control binding sites with cells.

### Hydrophobic Effect

With the unique internal hydrophobic and external hydrophilic structure of the phospholipid bilayer on the cell membrane, the hydrophobicity of AuNPs can significantly regulate the cellular interaction [98]. For hydrophobic AuNPs, the large sized AuNPs (> 10 nm) can enter the cell through an active process [99, 100], but the small ones can be hindered and trapped by the phospholipid bilayer. Baulin et al. conducted more detailed experimental and theoretical studies on the cell membrane interaction between hydrophobic AuNPs functionalized with dodecanethiol ligands with sizes of 2, 4 and 6 nm, respectively [101]. The results showed that 6 nm AuNPs could spontaneously translocate the bilayer within milliseconds, while both 2 and 4 nm AuNPs were trapped within the bilayer. To achieve rational utilization of hydrophobicity on the ultrasmall AuNPs, Zheng et al. increased the hydrophobicity on the surface of hydrophilic glutathione-coated AuNPs (GS-AuNPs, ~ 1.8 nm) with hydrophobic octanethiol through ligand exchange, and investigated the impact of partial hydrophobicity of cellular interactions of ultrasmall AuNPs (Fig. 3b) [102]. After ligand exchange, the formed amphiphilic AuNPs exhibited higher affinity for the cell membrane with an increased fluorescence intensity of 18 times. The hydrophobicity induced van der Waals forces overcame the electrostatic repulsion of GS-AuNPs, enhancing the affinity to the cell membrane and promoting cellular uptake dynamics.

The increased hydrophobicity of ultrasmall AuNPs provides a facile pathway for improving the cellular interactions of ultrasmall AuNPs. However, hydrophobic AuNPs can activate the innate immune system. Rotello et al. quantified the relationship between the hydrophobicity of ultrasmall AuNPs (~ 2 nm) and immune response [103]. A series of ultrasmall AuNPs with log P values, the hydrophobic values of the headgroups, ranging from 0.63 to 5.35. At the cellular level, hydrophobicity was essentially positively correlated with immune response. At the in vivo level, when the log P value is less than 1.95, an increase in hydrophobicity led to an increased immune response. However, when the log P value reached 3.77, the dependence of hydrophobic immunogenicity becomes less evident due to the poor biodistribution of highly hydrophobic AuNPs. Hydrophobic ultrasmall AuNPs can cause cellular toxicity. Chompoosor et al. investigated the relationship between hydrophobicity and cellular toxicity of ultrasmall AuNPs [104]. They synthesized a series of hydrophobic ultrasmall AuNPs (~ 2 nm) after a quaternary ammonium functionalization with a systematically varied hydrophobic alkyl tail (C1–C6). As the number of alkyl groups increased from C1 to C6, the IC50 value decreased from 6 to 0.71 μM. An increase of hydrophobicity enhanced the interaction of ultrasmall AuNPs with the phospholipid bilayer on the cell membrane. However, the high hydrophobicity can also cause toxicity and activate immunogenicity. Therefore, a balanced hydrophobicity should be considered to elicit immune responses and cellular toxicity of ultrasmall AuNPs before the biomedical applications.

### Surface Functionality Effect

**Fig. 4:**
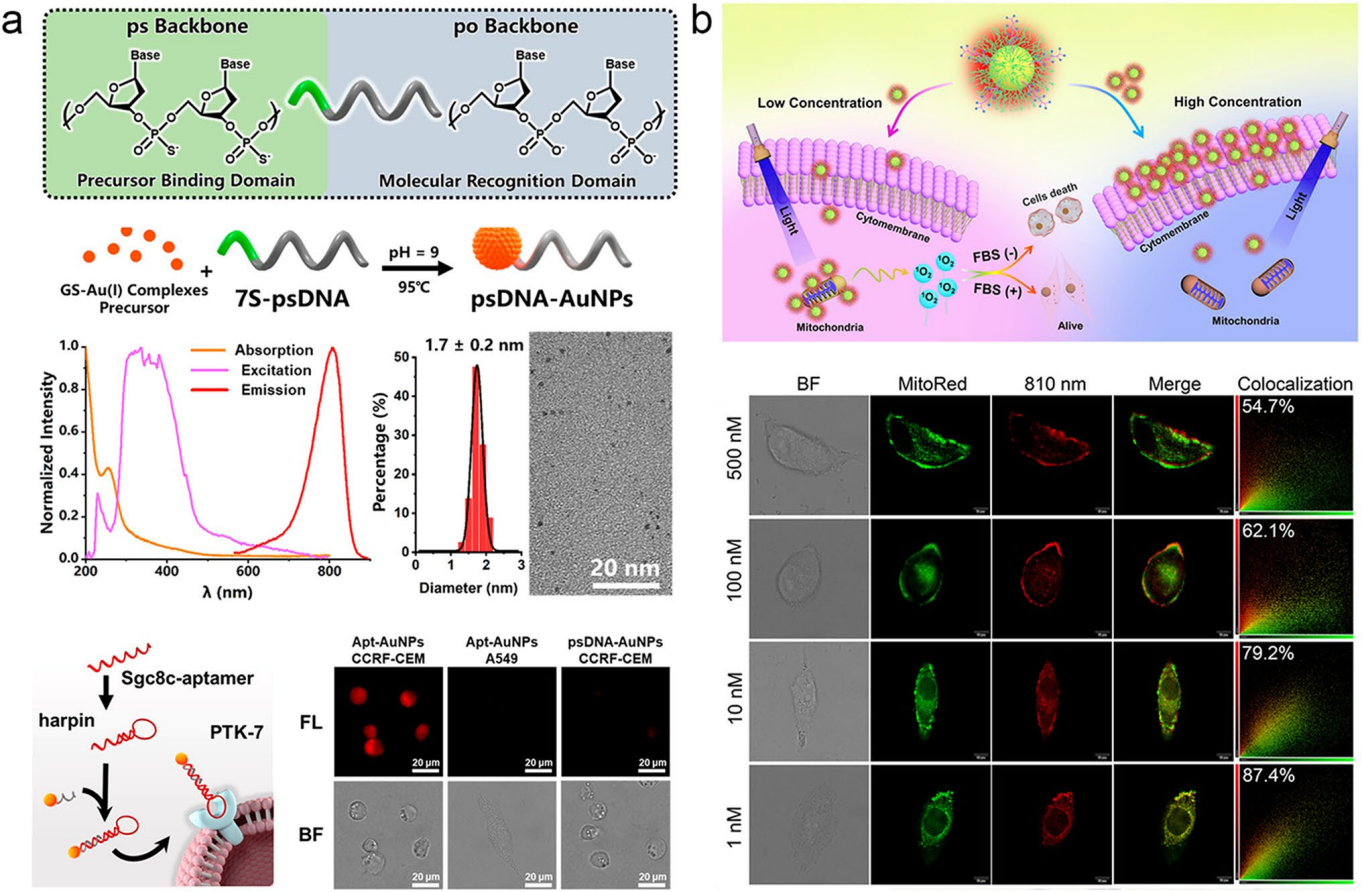
Effect of surface functionality and concentration on cellular interactions of ultrasmall AuNPs. **a** Apt-AuNPs with strict DNA valence to binding specific PTK-7 proteins [111]. Copyright (2020), American Chemical Society. **b** Concentration–dependent subcellular distribution of ultrasmall AuNPs [119]. Copyright (2020), John Wiley and Sons

With the sharply shrunken size, surface functionalization of the ultrasmall AuNPs is extremely important in the enchantment of cellular interactions in a selective manner toward different types of cells, and further change subcellular distribution. Currently, various surface functionalization strategies have been reported, such as the bioconjugation of targeted peptide [105–107], DNA [108], and antibodies [109, 110]. In our group, by taking advantages of phosphorothioates (ps)-modified DNA (psDNA) as a template [111], we reported a facile strategy in the in situ controlling the surface functionalization of NIR-emitting AuNPs (1.3 to 2.6 nm) with a discrete number of DNA (e.g., 1 and 2) (Fig. 4a). After hybridization with the sgc8c aptamer (Apt-AuNPs) that targets PTK7 proteins, overexpressed on the membranes of CCRF-CEM cells, the Apt-AuNPs showed significantly specific targeting ability towards CCRF-CEM cells over the human A549 cells unexpressed PTK7 proteins. Zhu et al. synthesized a methionine-functionalized ultrasmall AuNPs (Met-AuNPs, ~ 2.3 nm) for selective targeting of the overexpressed L-type amino acid transporter protein in A549 tumor cell over the WI-38 cells [112]. In their subsequent work, they introduced a lipophilic cation (4-mercaptobutyl) triphenyl phosphonium bromide (MTPB) onto Au_18_SG_14_ to realize the ligand-regulated subcellular distribution in mitochondria from lysosome [113].

### Concentration Effect

The investigation of the concentration-dependent biological behavior at the cellular level is also of great importance towards reduced the systematical toxicity [114–118]. The concentration variation can change the cellular interactions of ultrasmall AuNPs. In our group, we discovered that the NIR-emitting AuNPs co-coated with both GSH and cell-penetrating peptide CR8 (CR–AuNPs) showed a concentration-dependent subcellular distribution (Fig. 4b) [119], which exhibited a strong membrane-binding at high concentration (e.g., > 100 nM) but more endocytosis for mitochondria targeting at the low concentration region (e.g., < 10 nM). As a result of the concentration-dependent subcellular distribution, the ultrasmall CR–AuNPs showed photocytotoxicity in the relative low concentration region (e.g., 1 nM) after the light irradiation to generate singlet oxygen (^1^O_2_). This discovery facilitated the fundamental understanding of the concentration-dependent cellular interactions and potential cytotoxicity of ultrasmall AuNPs for future diagnosis and treatment. The fundamental understanding of the cellular interactions of ultrasmall AuNPs in more details can provide an insight towards the complicated interactions of AuNPs upon the living organisms, facilitating their clinical translation.

## Organ Interactions

When the nanoparticles are administrated into the body, most nanoparticles cannot transport to the intended disease tissue and are sequestered by the reticuloendothelial system (RES) organs (e.g., liver and spleen) or eliminated though the kidneys [120, 121]. The liver acts as a major RES organ that sequesters most (up to 90% ID) of the administered plasmonic AuNPs (> 6 nm) from bloodstream [122, 123]. Kidneys are a major organ for blood filtration and clearance of small AuNPs (e.g., < 6 nm), which also play a key role in governing the transport and clearance [124, 125]. A comprehensive understanding of the nanoparticle–liver interaction and nanoparticle-kidney interaction provides an overview of recent strategies on the precise control of off-target nanoparticle clearance to enable longer blood circulation and enhance transport in the target tissues.

### Kidney Interaction

**Fig. 5:**
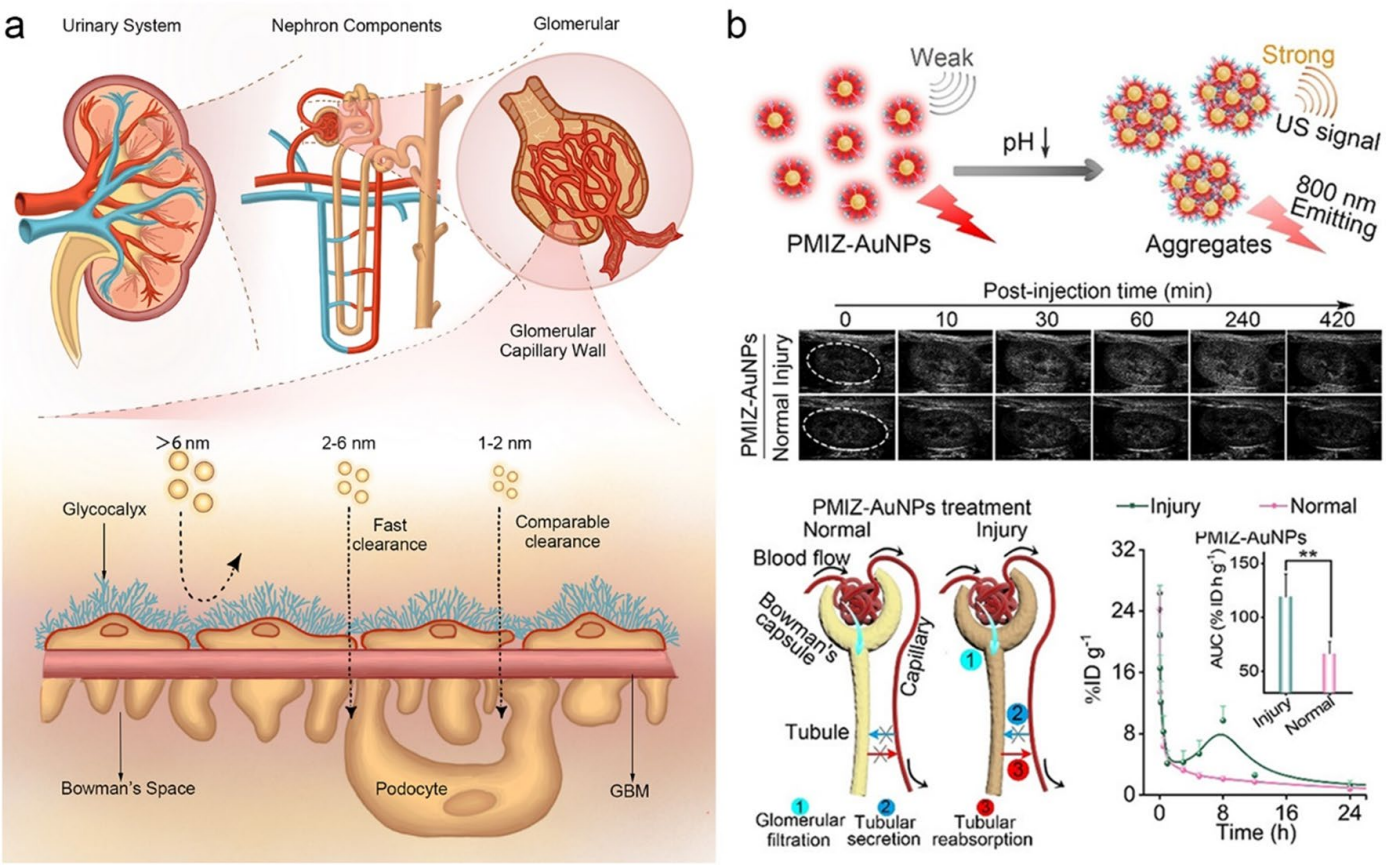
Mechanisms of kidney interaction. **a** Schematic diagram of the kidney structure and the corresponding glomerular filtration of AuNPs with different sizes. **b** The tubular reabsorption of the renal-clearable AuNPs in acidic kidneys [136]. Copyright (2021), John Wiley and Sons

When the ultrasmall AuNPs enter the kidneys through bloodstream, they are firstly filtered through glomerulus, followed by entering the proximal tubules [126, 127]. Then the ultrasmall AuNPs pass through the Henle loop, distal tubules and collecting ducts, and finally excrete through ureter into the bladder (Fig. 5a) [128]. The glomerular filtration barrier, as a “size cutoff” slit [129], retains larger nanoparticles (e.g., > 6 nm) in the body, while the ultrasmall AuNPs with sizes < 3 nm can be rapidly excreted through the kidney’s glomerular filtration. Zheng et al. discovered that in the sub–nanometer size regime [130], the glomerular filtration barrier could serve as an atomically precise “bandpass” filter, significantly slowing down the clearance rate of the few-atom AuNCs (e.g., Au_18_, Au_15_ and Au_10–11_). The renal clearance rates of Au_18_, Au_15_, and Au_10–11_ were 10.79%, 6.03%, and 5.29% injection dose (ID), respectively, significantly low than those of Au_25_ early elimination stage (within 2 h post-injection (p.i.), 46.71% ID), which indicated that only a few-atom decrease of the AuNCs resulted in a 4–9 times reduction in renal clearance rate. It was then found that the smaller AuNCs were readily trapped by the glomerular glycocalyx rather than the larger AuNPs. This glomerular glycocalyx interaction of sub-nanometer AuNCs slowed down the extravasation from normal blood vessels and enhanced the passive targeting to tumors through the EPR effect. This discovery demonstrates the size precision in the nanoparticle–kidney interaction, bringing forward guidance to develop nanomedicines for many diseases such as the glycocalyx dysfunction.

The filtered AuNPs then flow from the Bowman space into the proximal tubules (PTs) [131–133]. PTs are covered with a dense brush border composed of negatively charged microvilli that extend into the tubular cavities, governing the subsequent active uptake, reabsorption, and metabolism of the filtered AuNPs. The proximal tubular epithelial cells (PTECs) can transport AuNPs from the blood into the tubular lumen and urine through transporter-mediated influx- and efflux-processes. Zheng et al. discovered an unrecognized mechanism for eliminating ultrasmall AuNPs utilizing mitotically quiescent PTECs [134]. Upon the PEGylated AuNPs (~ 2.6 nm) entering the PTs, the ultrasmall AuNPs were internalized by PTECs through endocytosis. These endocytosed AuNPs could even partially transform into large nanoassemblies (200–300 nm) inside the lysosomes/endosomes. By squeezing the balloon -like extrusions (~ 5 µm) through dense microvilli, the intact endocytosed AuNPs were transported into the extrusions along with other organelles and then pinched off the extrusions from the cell membrane into the lumen. Within a month, PTECs re-eliminated > 95% of the endocytosed AuNPs and nanoassemblies into the urine. This organelle–extrusion mechanism represents a new nanoparticle–elimination pathway in the kidneys, which is also an intrinsic “housekeeping” function of normal PTECs to self-renew intracellular organelles.

### Interaction of Injured Kidney

Kidney disease is one of the great threats to health, but it is difficult to differentiate using the routine clinical markers (e.g., blood urea nitrogen and creatinine) at its early stages, resulting in late-stage diagnosis and lack of early intervention [128, 135]. Typically, renal-clearable ultrasmall AuNPs are rapidly excreted into urine after glomerular filtration [64], which show relatively weak interaction with the renal compartments. Renal tubular cells (RTCs) are the primary target for kidney injury and disease progression. In our group, we designed luminescent PEGylated AuNPs co-coated with a pH-responsive zwitterionic imidazole group (1.9 nm) [136], which exhibited charge-reversal and aggregation capabilities in acidic kidney microenvironments. The synthesized AuNPs showed a charge–reversal ability from negative charge (− 10.9 ± 1.0 mV) to positive charge (17.4 ± 1.6 mV) with pH decreased from pH 7.4 to pH 5.5. In addition, the HD of AuNPs was 3.5 ± 0.4 nm at pH 7.4, but significantly increased to 1048.7 ± 225.7 nm at pH 5.5. In an acidosis-induced early kidney injury model, the acidic kidney environment enhanced the reabsorption of AuNPs, allowing more AuNPs to in situ aggregate in the RTCs. The prolonged retention of AuNPs in the injured kidney provided both enhanced ultrasound and fluorescence signals for noninvasively imaging of kidney injury with precise anatomical information. The discovery of tubular reabsorption of the renal-clearable metal nanoparticles in the acidic kidneys opened a new pathway for early diagnosis and treatments of specific renal diseases (Fig. 5b).

### Liver Interaction

**Fig. 6:**
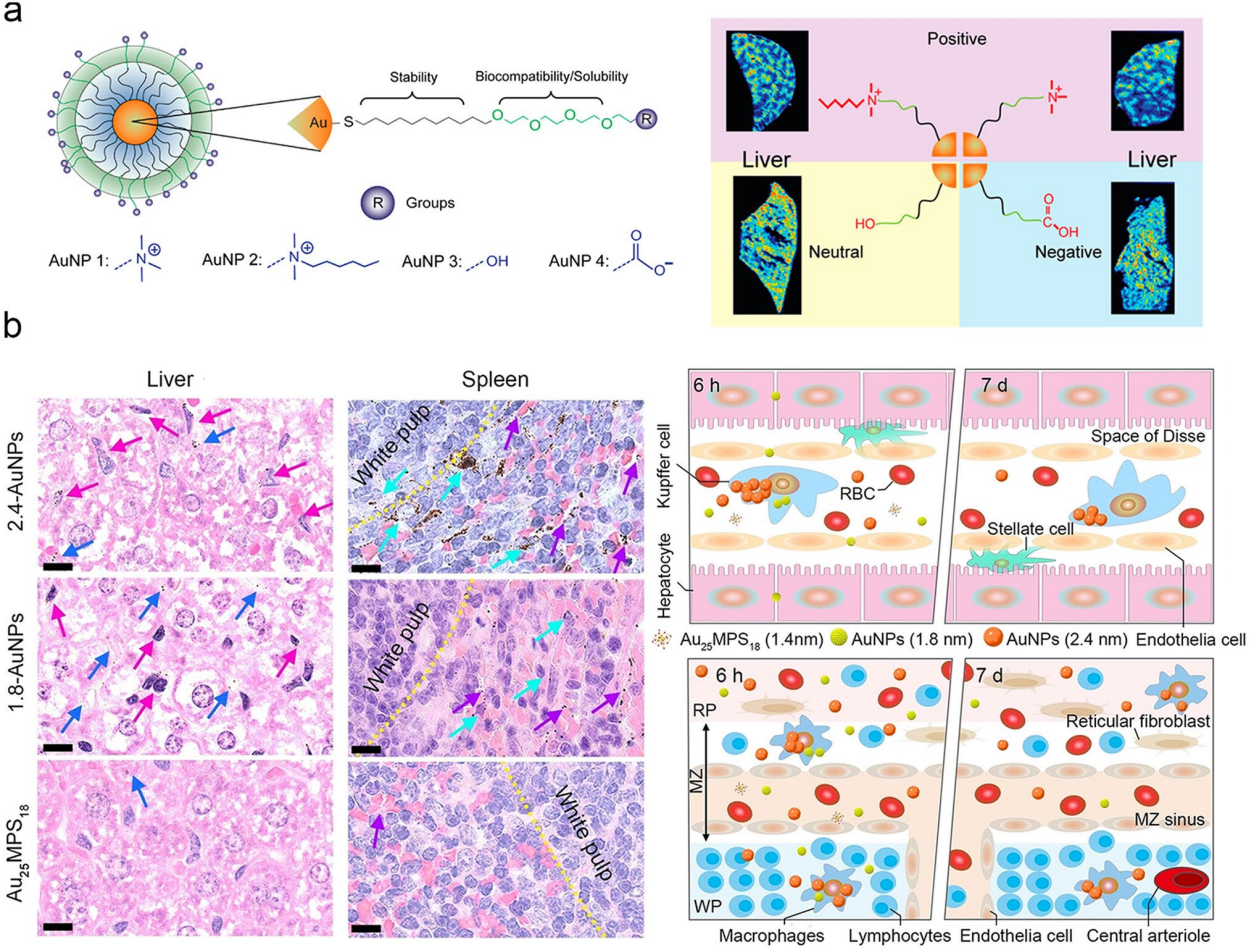
Effects of ultrasmall AuNPs on the sub–organ distribution. **a** Surface charge controls the sub-organ biodistributions of ultrasmall AuNPs in liver [124]. Copyright (2016), American Chemical Society. **b** Precise size-dependent sub–organ distribution in liver and spleen [141].Copyright (2023), John Wiley and Sons

Liver is the largest solid organ for detoxification in living body [137]. Normal liver sinusoidal endothelial cells typically show fenestrations of 50–200 nm in diameter [138], favorable for the extravasation of ultrasmall AuNPs (< 3 nm) before the entrance of the space of Disse. The entered AuNPs interact with hepatocytes and then are transported through transcytosis into the bile ducts, ultimately being eliminated from the body through the intestines [139]. Understanding the liver interaction of ultrasmall AuNP is critical to the utilization of ultrasmall AuNPs in the disease diagnosis and treatment. Surface charge plays an important role in governing the liver interaction of ultrasmall AuNPs. The surface charge effect on the liver inter on biological distribution is explored. Vachet et al. investigated the sub–organ distributions of different charged ultrasmall AuNPs (2 nm) including positively charged, negatively charged and neutral ones (Fig. 6a) [124]. It was found that positively charged AuNPs accumulated in hepatocytes and endothelial cells, while the neutral and negatively charged ones exhibited a more widespread distribution throughout the liver (at 24 h p.i.). The further quantitative analysis of the Au element signals in live tissue slices demonstrated that positively charged AuNPs distributed in a more heterogeneous pattern than those of the negatively charged or neutral ones. Therefore, surface charge is a crucial factor in determining the nanoparticle-liver interactions. Besides the influence of charge on the liver interaction of ultrasmall AuNPs, variations within the sub-nanometer regime (~ 0.5 nm) also affect the liver interaction of ultrasmall AuNPs. Although ultrasmall AuNPs can be quickly eliminated through the kidneys, the RES system (e.g., liver and spleen) serves as a size-dependent barrier in the removal of ultrasmall AuNPs from the bloodstream [140]. In our group, we investigated the size-dependent sub-liver distribution of ultrasmall AuNPs in the sub-nanometer regime (Fig. 6b) [141]. We firstly developed a in situ synergetic synthesis and separation strategy to achieve large–scale atomically precise Au_25_MPS_18_ (MPS = sodium 3-mercaptopropanesulphonate) and AuNPs with few sub–nanometer differences (from 2.4 to 1.8 nm and finally to 1.4 nm). We discovered that the 2.4-AuNPs (with sizes of 1.4–2.4 nm) distributed heavily throughout the liver (e.g., hepatic Kupffer cells and hepatic sinusoids) and spleen (e.g., splenic macrophages both in the red pulp and white pulp, splenic venous sinus) at 6 h p.i. The 1.8-AuNPs (with sizes of 1.4–1.8 nm) mainly appeared in the hepatic sinusoids and splenic venous sinus. However, the atomically precise Au_25_MPS_18_ with a size of 1.4 nm were hardly found in the liver or spleen. Further investigation revealed that a sub–nanometer difference in size would significantly increase the elimination rates of the ultrasmall AuNPs in liver and spleen. Therefore, the in vivo phagocytosis and traps of ultrasmall AuNPs in the hepatic Kupffer cells and splenic macrophages were a precisely size-dependent in the sub-nanometer regime.

**Fig. 7:**
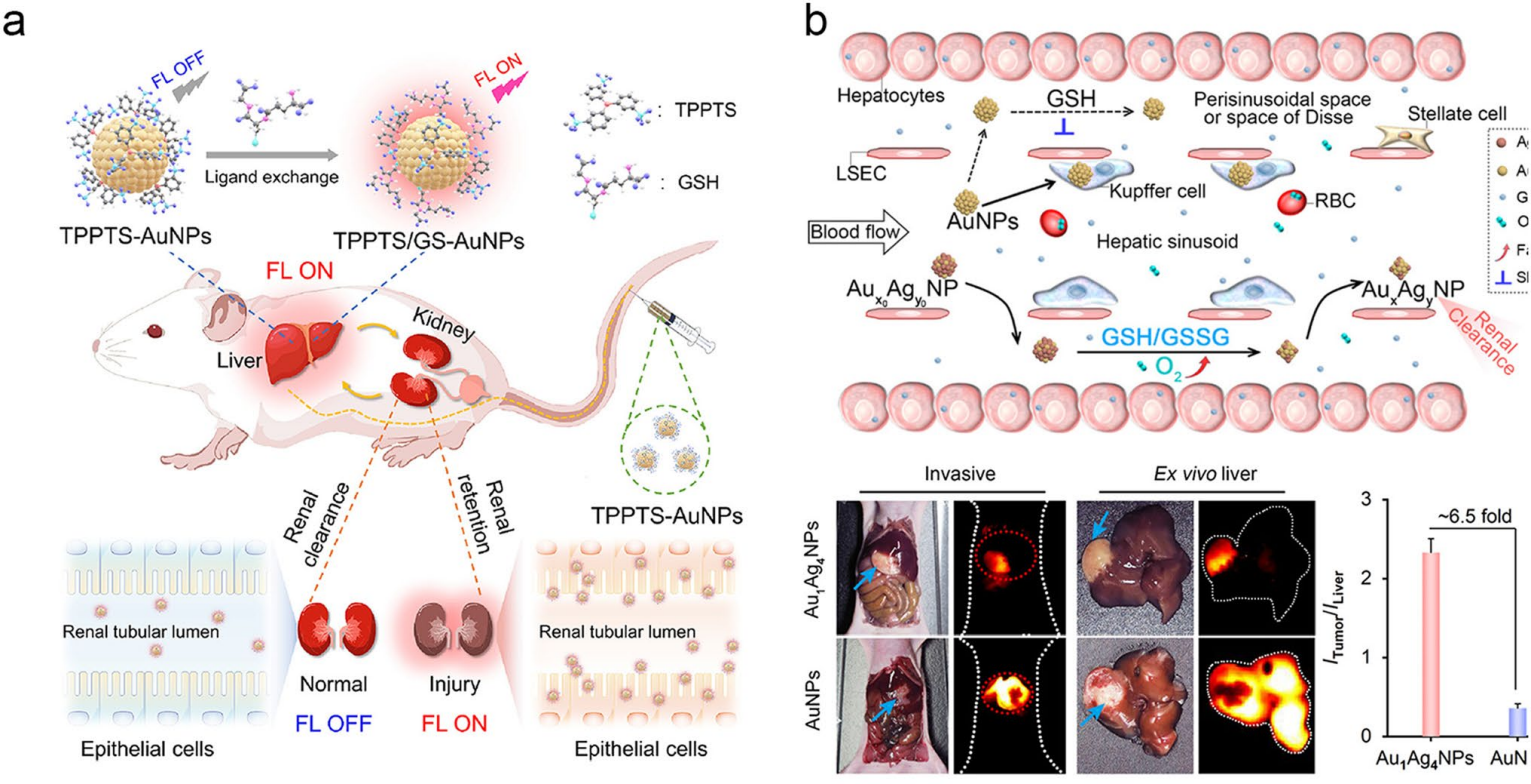
Mechanism of liver interaction with ultrasmall AuNPs. **a** GSH-activated emission of ultrasmall AuNPs for early kidney injury diagnosis [144]. Copyright (2022), American Chemical Society. **b** Rapid biotransformation of ultrasmall bimetallic nanoparticles in hepatic sinusoids [145]. Copyright (2023), American Chemical Society

In addition to being sequestered in the liver, ultrasmall AuNPs can also be biotransformed by the abundant GSH in liver. Zheng et al. conducted a research on the GSH-mediated detoxification mechanism of ultrasmall AuNPs [142]. They designed a thiol-activatable fluorescent nanoprobe, ICG_4_–GS–Au_25_ (ICG = indocyanine green), which can bind serum proteins and transport to liver sinusoids. The ICG emission was quenched due to the electron transfer between ICG and Au_25_, but the ICG fluorescence turned on instantly after the detachment of ICG under the activation of GSH. With this principle, the in vivo liver biotransformation kinetics was non-invasively imaged. It was demonstrated that glutathione efflux from hepatocytes led to concentrated glutathione and cysteine in liver sinusoids to transform surface chemistry of the nanoparticles, which significantly increased the resistance to serum protein absorption and changed the blood retention, targeting and clearance. In their subsequent work, they utilized ICG_4_-GS-Au_25_ probe to non-invasively monitor the GSH depletion in liver through GSH-mediated conversion [143]. A linear relationship between fluorescence intensity and GSH concentration was constructed, and the depletion of liver GSH could be detected through both fluorescence imaging and blood testing. In our group, with the abundant GSH in liver sinusoids, we developed a facile strategy to activate the emission of ultrasmall AuNPs (~ 1.4 nm) with low background for imaging of early kidney injury (Fig. 7a) [144]. Quantitatively activated emission at ∼ 1026 nm was achieved from the ligand exchange of triphenylphosphine–3,3′3–trisulfonic acid (TPPTS)–coated AuNPs with GSH. The in vivo GSH-exchanged AuNPs show enhanced interactions with acidic renal tubular epithelial cells, which resulted in noninvasive monitoring of acidosis-induced early kidney injury with both high sensitivity (contrast index, ~ 3.9) and long-time imaging window (> 6.5 h).

Liver sequestration is one of the main obstacles to the efficient nanoparticle transport to the disease sites [146]. To avoid the phagocytosis of nanoparticle in liver, in our group, we designed ultrasmall luminescent gold–silver bimetallic NPs (Fig. 7b) [145], which could be fast transformed in hepatic sinusoidal microenvironment with abundant both GSH and oxygen (pO_2_ =  ~ 44.4 mmHg). With the help of the active silver atoms, the gold–silver bimetallic NPs (~ 2.3 nm) transported quickly into the liver right after iv injection, and then synergistically reacted with oxygen and GSH in the hepatic sinusoid to reduce the serum protein binding. Due to the rapid biotransformation in liver, the gold–silver bimetallic NPs traveled from liver back into the blood and finally cleared out of body through renal clearance. In sharp contrast, most of the monometallic AuNPs were rapidly sequestrated by Kupffer cells as a result of the slow biotransformation. The rapid sinusoidal biotransformation of gold–silver bimetallic NPs avoided the phagocytosis in liver, which significantly prolonged the blood circulation and further enhanced the targeting efficiency at the disease site. The signal–to–noise ratio (S/N) of tumor to liver tissue for gold–silver bimetallic NPs was ~ 2.3, which was ~ 6.5 times higher than that of the monometallic AuNPs. It is very interesting to note that the liver sequestration can be turned into a beneficial nanomedicine storage with the fast biotransformation in sinusoids for manipulating the transport efficiency to disease site.

## Tumor Interactions

The development of nanoparticles provides new strategies for cancer diagnosis and treatment [147, 148]. Compared to the small molecules, nanoparticles have a longer blood circulation time, which increases their retention in disease sites via EPR effect [149, 150]. However, significant accumulation of nanoparticles in RES organs (e.g., liver, spleen and bone) also can lead to long–term toxicity risk, resulting in low targeting specificity in disease sites [151, 152]. Tumors are structurally heterogeneous with nonuniformly leaky vasculature and dense interstitial structures that hinder the deep penetration of large nanoparticle into tumor tissue, especially in areas distant from the vasculature. Ultrasmall AuNPs can effectively address the challenges of fast distribution and low penetration from the large nanoparticles [153, 154], which also combines both of the advantages of small molecules (e.g., efficient renal clearance and low nonspecific tissue accumulation) and conventional nanoparticles (e.g., long blood circulation and EPR effect). In addition, as compared with other ultrasmall inorganic nanoparticles including quantum dots and semiconductor oxides (Table 1), the ultrasmall AuNPs were highly stable during the blood circulation system, which showed faster and more efficient renal clearance with lower nonspecific accumulation in the heathy liver and spleen. Furthermore, it was found that the ultrasmall AuNPs exhibited a more satisfactory balance between the tumor-targeting efficiency and rapid clearance, providing great clinical translation opportunities into the in vivo disease imaging and treatment.

**Table 1:** Comparison of biodistribution and tumor targeting efficiencies of the typical ultrasmall inorganic nanoparticles

Nanoparticles	Surface ligand	Core/HD (nm)	Time of p.i. (h)	Urine (%)	Biodistribution (% ID g^−1^)			Refs.
					Liver	Spleen	Tumor	
GS-AuNPs	GSH	1.7/2.1	24	50.5	3.7 ± 1.9	0.3 ± 0.1	NR	[155]
GS-[^198^Au] AuNPs	GSH	2.6/3.0	24	> 40	3.0 ± 0.5	1.8 ± 0.3	NR	[156]
CY-PSMA-1-Au_25_ NCs	PSMA^a^	1.5/3.0	4	> 20	< 3	< 53	8.9	[157]
Au@DTDTPA	DTDTPA^b^	2.4/6.6	24	> 60	< 10	< 2	< 3	[158]
02PMIZ-AuNPs	02PMIZ^c^	1.7/4.2	6	> 50	< 3	< 3	6.9	[159]
PEG-AuNPs	PEG	2.3/5.5	12	> 30	< 5	< 5	8.3	[160]
GS-Au NCs	GSH	1.5/2.4	48	NR	< 30	< 5	> 12	[161]
InAs/InP/ ZnSe QDs	MPA^d^	NR	4	NR	42.6	10.3	11.0	[162]
Ag_2_S QDs	BSA^e^	2.1/NR	24	NR	20	> 5	NR	[163]
Cu_2-x_S NDs	PEG	< 5/10	8	3(48 h)	> 50	> 35	3.6	[164]
Ti_2_N QDs	SP^f^	4.8/NR	4	NR	> 20	< 3	> 10	[165]
Ag_2_S QDs	GSH	4.2/NR	24	NR	> 5	> 5	< 1	[166]
Bi NPs	PEG	3.8/NR	24	NR	> 17	> 28	6.26	[167]
WO_3−x_ NPs	NR	1.1/7	4	NR	> 14	> 13	< 3	[168]
PVP-ZrC NDs	PVP^g^	5/NR	24	> 50%	> 15	> 12	< 10	[169]

*NR* not reported

^a^Prostate specific membrane antigen

^b^Diethylenetriaminepentaacetic acid

^c^MSA-SH:PEG-SH = 0.2:3

^d^Mercaptopropionic acid

^e^Bovine serum albumin

^f^Soybean phospholipid

^g^Polyvinylpyrrolidone

### Passive Targeting

**Fig. 8:**
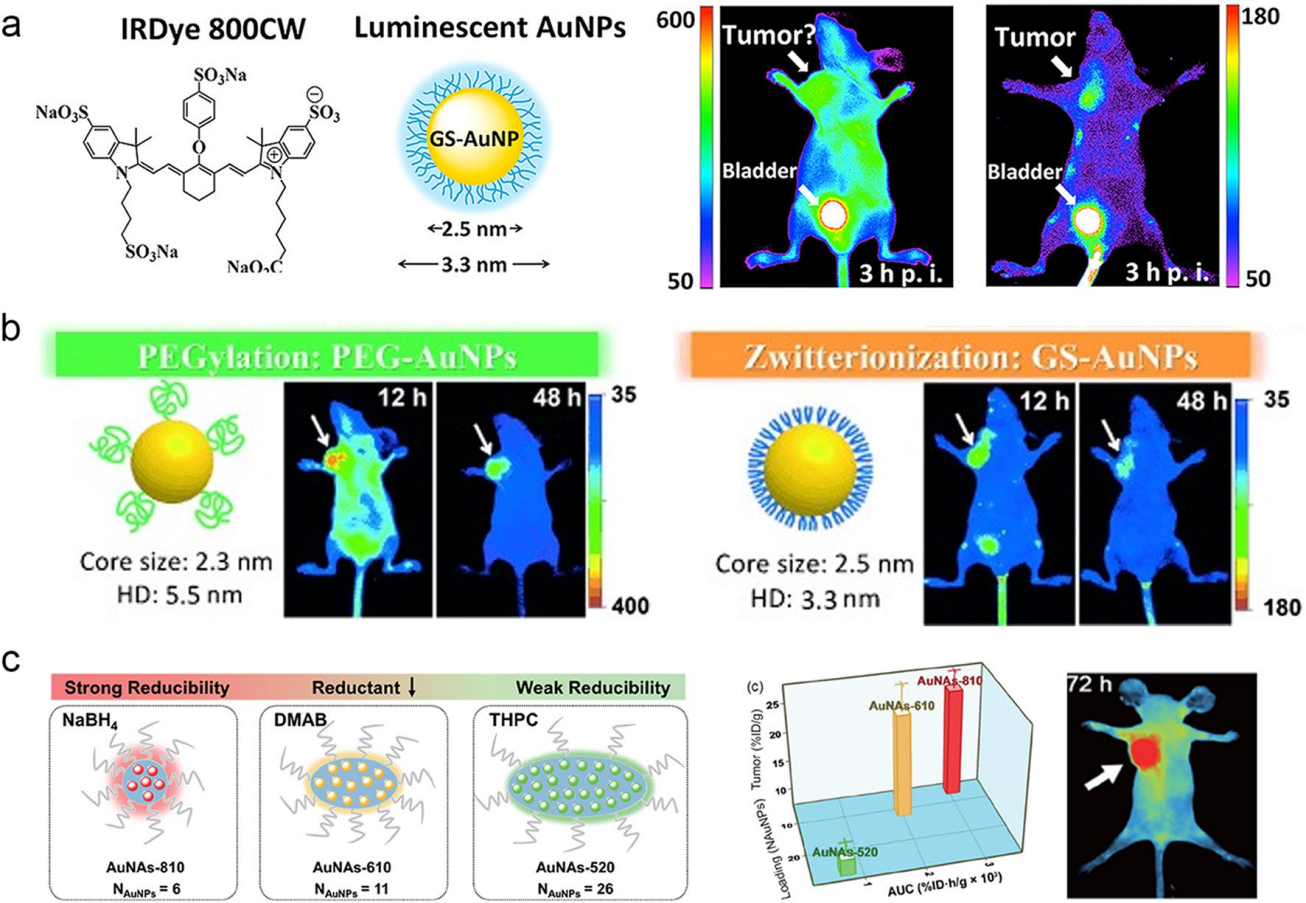
Passive tumor targeting of ultrasmall AuNPs. **a** Passive tumor targeting of renal–clearable GS-AuNPs [170]. Copyright (2013), American Chemical Society. **b** PEGylation and zwitterionization in the tumor targeting of ultrasmall AuNPs [160]. Copyright (2013), John Wiley and Sons. **c** Well-controlled gold nanoassemblies for efficient tumor targeting [177]. Copyright (2020), Springer Nature

The ultrasmall AuNPs exhibit fast renal clearance and two-compartment pharmacokinetics like small molecules. Whether the ultrasmall AuNPs retain the EPR effect, a unique feature from conventional nanoparticles in passive tumor targeting, is critical to the future biomedical applications. To unveil the EPR effect of ultrasmall AuNPs, using zwitterionic GSH as surface ligand to minimize nonspecific RES uptake, Zheng et al. synthesized renal-clearable ultrasmall GS-AuNPs (~ 2.5 nm) and conducted a detailed comparison in the passive tumor targeting with an organic fluorophore, IRDye 800CW [170]. They discovered that the ultrasmall GS-AuNPs retained in the tumor at a concentration 10 times higher than the dye molecules (12 h p.i.), but the clearance rate from normal tissues is 3 times faster than that of dye molecules (Fig. 8a). The much longer tumor retention time and faster normal tissue clearance of ultrasmall AuNPs, indicated that the well-known EPR effect indeed existed ultrasmall AuNPs. The GS-AuNPs (~ 2.6 nm) were further demonstrated to across the blood–brain barrier in the passive targeting of gliomas. By comparing the transport efficiency of 2.6-nm GS-AuNPs and 18-nm GS-AuNPs in glioma, the 2.6-nm GS-AuNPs accumulated in glioma via EPR effect with a targeting efficiency (0.2 ± 0.04%ID/g) of 2.3 times higher than that of the 18-nm AuNPs (0.08 ± 0.05%ID/g). The stronger EPR effect of 2.6-nm GS-AuNPs as compared to 18-nm GS-AuNPs was attributed to the higher vascular leakage of ultrasmall AuNPs to enter the glioma interstitium for a longer retention time in gliomas. Since GSH contains more than one anchoring groups (e.g., –SH, –COOH and –NH_2_) towards the gold surface, in our group, we demonstrated that not only the strong anchoring site of S-Au, but also the weak anchoring sites from N-Au and COO-Au showed significant influences to the passive tumor targeting of GS-AuNPs [171]. The more anchoring sites of COO-Au and more exposed surface –NH_2_ led to prolonged blood circulation and passive tumor targeting efficiency (5.1 ± 0.6%ID g^−1^), which was more than 4.4 times than those of more anchoring sites of N-Au and more exposed –COOH (1.2 ± 0.08%ID g^−1^). These results indicated the significance of the weak anchoring sites in the surface functionalization of nanoparticles.

PEGylation of nanoparticle is commonly used to reduce the nonspecific accumulation in organs and prolong the blood circulation [172, 173], which enhances the EPR effect in the passive tumor targeting efficiency. Zheng et al. compared the passive tumor targeting of PEGylated AuNPs (~ 2.3 nm) with the renal–clearable zwitterionic GS-AuNPs (Fig. 8b) [160], and found that the tumor targeting efficiency of PEGylated AuNPs was 3 times higher than that of the zwitterionic ones. The high tumor-targeting efficiency of the PEGylated AuNPs was attributed to enhanced EPR of PEGylated AuNPs relative to zwitterionic AuNPs in the increased blood retention. Subsequently, a series of PEGylated ultrasmall AuNPs with a strong EPR effect were reported. Wu et al. utilized the miniprotein Min-23 as a template to synthesize Min − 23@AuNCs (1.8 nm) [174]. After the PEGylation, the pharmacokinetics of PEGylated Min − 23@AuNCs showed both extended distribution half-life (from 14.9 to 18.8 ± 1.7 min) and elimination half–life (from 3.1 to 6.3 ± 0.8 h), which greatly increased the transport to the tumors. Recently, commercial amphiphilic block copolymer (e.g., pluronic F127) with the PO hydrophobic core surrounded by hydrophilic PEG block surface was also developed as capping agent for the ultrasmall AuNPs to extend the blood circulation time and enhance the passive tumor targeting efficiency [175, 176]. In our group, using an amphiphilic block copolymer (ABC) pluronic F127 as template, we developed a straightforward strategy for in situ fabrication of well–controlled gold nanoassemblies with ultrasmall AuNPs (1.7 nm) encapsulated inside the hydrophobic core (Fig. 8c) [177]. The formed nanoassemblies showed long blood retention with tumor targeting efficiency as high as ~ 25.3%ID g^−1^. Therefore, the well-designed PEGylation in the functionalization of ultrasmall AuNPs can greatly enhance their passive tumor targeting efficiency and transport to the disease sites.

### Active Targeting

The EPR effect can guide ultrasmall AuNPs transport into the tumor site with the blood flow. In order to increase the targeting efficiency of ultrasmall AuNPs at the tumor site and reduce the non-specific accumulations in healthy organs, various active targeting strategies have been advanced [178]. According to the chemical surface functionalization strategies, the active targeting pathways of ultrasmall AuNPs can be divided into the following categories: receptor-mediated targeting, peptide-mediated targeting, antibody-mediated targeting, and aptamer-mediated targeting [147, 179].

#### Receptor-mediated Targeting

**Fig. 9:**
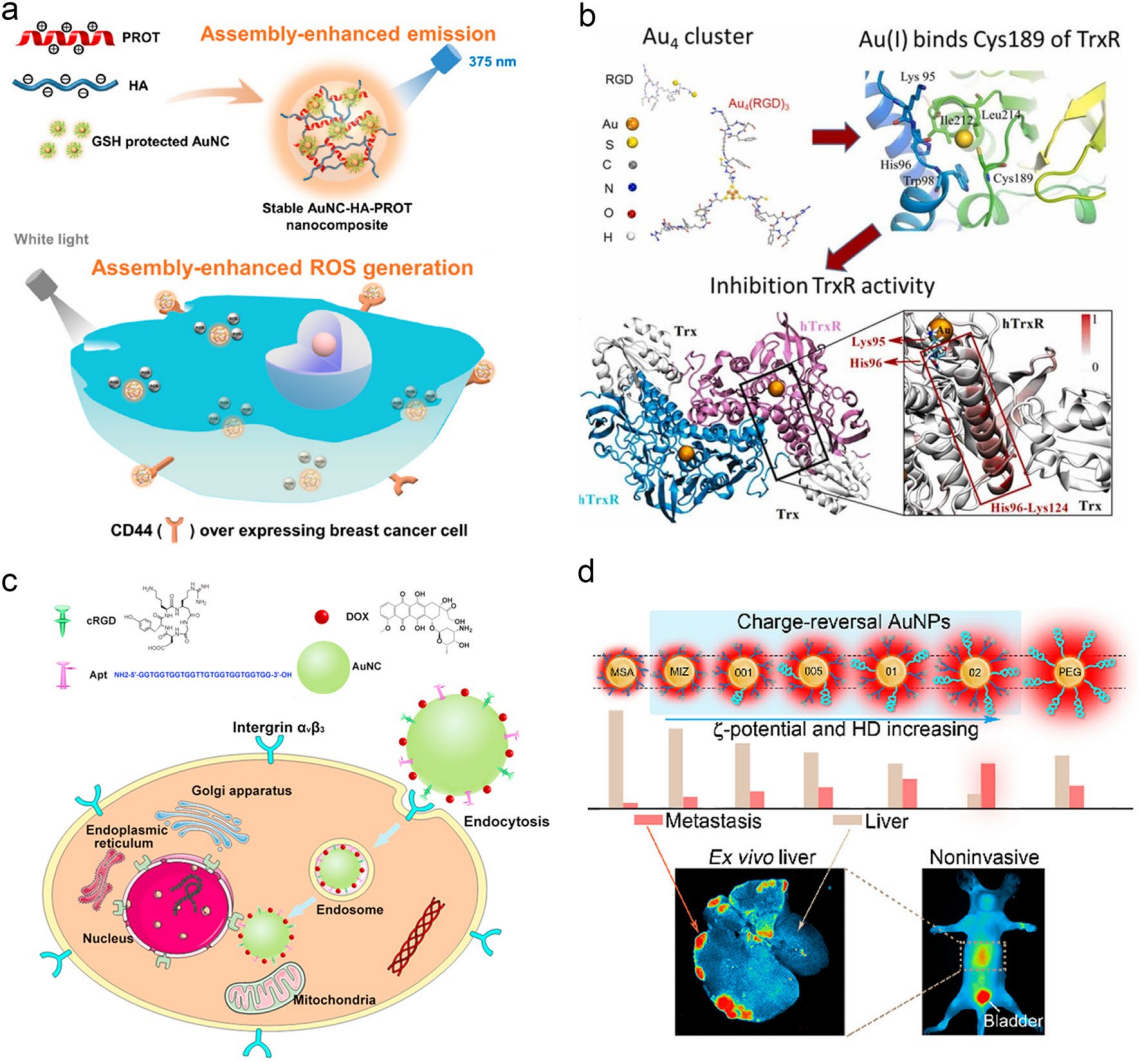
Active tumor targeting of ultrasmall AuNPs. **a** AuNC-HA-PROT nanocomposites targeting CD44 antigens were used for cell imaging and therapy [182]. Copyright (2019), American Chemical Society. **b** Au_4_(RGD)_3_ inhibits human thioredoxin reductase activity via specifically binding of Au to Cys189 [191]. Copyright (2022), Elsevier. **c** Dual targeting luminescent AuNC-cRGD-Apt for tumor imaging and deep tissue therapy [199]. Copyright (2016), Elsevier. **d** Tumor-acidity activated charge-reversal of ultrasmall AuNPs to achieve highly tumor-targeting specificity [159]. Copyright (2020), American Chemical Society

Receptor-mediated targeting is a well-developed strategy to functionalize ultrasmall AuNPs in the active targeting of tumors by conjugation of ligands selectively binding to the overexpressed receptors on tumors [180, 181]. The widely investigated receptors for active targeting mainly involve hyaluronic acid (HA) receptors, transferrin (Tf) receptors, folate receptors and glucose transporters. HA [182, 183], main component of the extracellular matrix, is used to maintain the basic structure of cells, which can target the CD44 receptor, overexpressed on the tumor cell surface in the regulation of tumor angiogenesis and metastasis. Li et al. utilized both electrostatic and hydrophobic effects to embed GS–AuNCs (2.5 nm) into negatively charged HA and cationic protamine (PROT) to generate the AuNC-HA-PROT nanocomposites [182], which were used to target CD44 receptors overexpressed on MDA-MB-231 cells (Fig. 9a). Tf receptor is a cell membrane-associated glycoprotein [184], which is highly overexpressed on tumor cells with expression levels 100-fold higher than that of the average expression in normal cells. Yan et al. developed a Tf-functionalized AuNCs (Tf-AuNCs, 2.6 ± 0.5 nm)/graphene oxide (GO) nanocomposite (TfAuNCs/GO), which showed a turn-on NIR emission (710 nm) towards HeLa cells for in vivo tumor imaging [185]. Folate receptor is also highly overexpressed on the surface of cancer cells [186]. Tian et al. utilized BSA-protected AuNCs conjugated with folic acid for targeted imaging of FR^+ve^ HeLa cells [187]. Glucose transporters overexpressed in the cancer cells can guide glycoconjugated ultrasmall AuNPs enter the cancer cells via active transport mechanisms. In our group, using 1-thio-β-D-glucose as both the surface ligand and the reducing agent, we developed a facile in situ glycoconjugation strategy for the synthesis of NIR-emitting gold glyconanoparticles (AuGNPs, ~ 2.4 nm) [188], which showed both activity towards glucose transporters in cancer cells and prolonged blood circulation. The ultrasmall AuGNPs showed similar low nonspecific organ retention to that of the renal-clearable GS-AuNPs, but ∼ 10 and 2.5 times higher in vitro and in vivo tumor-targeting efficiencies, respectively. This in situ glucose functionalization of ultrasmall AuNPs not only enhances the tumor targeting efficiency but also reduces non–specific enrichment in healthy organs.

#### Peptide-mediated Targeting

During the tumor proliferation, new blood vessels are formed with integrin α_V_β_3_ highly overexpressed in tumor cells [189, 190]. The Arg-Gly-Asp peptide (RGD) can specifically bind to integrin α_V_β_3_, enabling active targeting of tumors. Xing et al. used cyclic RGD acid (cRGD) peptide as a template to synthesize AuNCs (1.7 ± 0.1 nm) (cRGD-AuNCs) for targeting α_V_β_3_ integrin–positive cancer cells [190]. The accumulation of cRGD-AuNCs at tumor site could reach 6.4 ± 1.3% ID (at 4 h p.i.). In addition, the cRGD-AuNCs could be not only as a fluorescent nanoprobe to stain α_V_β_3_ integrin-positive tumor cells but also a radiosensitizer for radiation cancer therapy. Gao et al. synthesized an Au_4_ cluster (Au_4_(RGD)_3_) functionalized with cRGD peptide [191]. This Au_4_ cluster firstly bound with human thioredoxin reductase (hTrxR) through both amino acid residues via electrostatic and van der Waals forces. Furthermore, the Au (I) from the metabolism of Au_4_ cluster specifically bound to the thiolate of Cys189 of hTrxR protein, resulting in the inhibition of hTrxR activity and cell apoptosis. This study indicated that the cRGD–AuNCs could inhibit hTrxR activity (Fig. 9b).

#### Antibody-Mediated Targeting

Antibodies can specifically bind to antigens to specifically target tumor tissues with enhanced therapeutic efficacy [192, 193]. The monoclonal antibodies (mAb) used to improve the targeting efficiency of nanoparticles have been widely reported. Trastuzumab (Herceptin) is a mAb that targets the extracellular domain of the ErbB-2 receptor overexpressed in breast cancer. Irudayaraj et al. synthesized fluorescent BSA-protected AuNCs (~ 2 nm) conjugated with Herceptin (AuNCs-Her) [194], which were used for specific targeting in ErbB2 overexpressed breast cancer towards both imaging and cancer therapy. Auguste et al. constructed a sensing nanoplatform with AuNCs-loaded liposomes after functionalization of ErbB2/Her2 antibody [195], which were used for amplified colorimetric detection of HER2-positive breast cancer cells. The peroxidase–like activity of the nanoplatform adsorbed on HER2-positive breast cancer cells could be used to quantitatively measure the HER2-positive breast cancer cells.

#### Aptamer-mediated Targeting

DNA aptamers are screened synthetic DNA oligonucleotides that can bind to various specific targets [196]. Surface functionalization of DNA aptamers endows nanoparticle with unique ability to specifically recognize various targets. Aptamer AS1411 is one of the tumor-targeted DNA aptamers [197, 198], targeting nucleolin protein located both on cancer cell surface and in nucleus. Chen et al. reported a nanoplatform conjugated AuNCs (~ 3.0 nm) with both cRGD and aptamer AS1411 (AuNC-cRGD-Apt) [199]. The combination of aptamer–mediated active targeting of tumor tissues and adapter-mediated targeting of the cytoplasm and nucleus was used for specific tumor targeting (Fig. 9c). MUC1 is an overexpressed transmembrane protein that associates with both inflammation and cancer growth. Wang et al. used the DNA MUC1 aptamers as a protective agent and targeted molecule to synthesize ultrasmall fluorescent AuNCs (1.5 ± 0.3 nm) [200], which effectively targeted overexpressed mucin on 4T1 tumor cells.

### Tumor Acidic Microenvironment Targeting

Different from the normal tissues, solid tumors exhibit unique microenvironments including dense and leaky microvasculature as well acidic extracellular pH values (pHe 6.5–7.2) [201–203]. Renal-clearable ultrasmall AuNPs with HDs smaller than 5.5 nm can permeate the dense and leaky tumor blood vessels with pore sizes of 300–1200 nm. Various “charge-reversal” strategies based on the tumor acidic microenvironmental stimuli (pHe 6.5–7.2) were reported to increase the accumulation and cellular uptake of nanoparticles in tumor site [204, 205]. Zheng et al. investigated how the tumor vasculature and local acidity affect the targeting and retention of ultrasmall AuNPs (~ 2 nm) [206]. Both GS–AuNPs without acidity targeting and GC-AuNPs with pH-dependent cellular membrane adsorptions were synthesized and further investigated their targeting efficiencies with two prostate cancer models: PC-3 tumors (pH 6.9, high microvascular densities) and LNCaP tumors (pH 6.5, low microvascular densities). After 24 h p.i., the accumulation of GC-AuNPs in LNCaP tumors (9.5 ± 2.2% ID g^−1^) was twice as high as that in PC-3 tumors (4.4 ± 0.65% ID g^−1^). However, the acidity effect on the tumor accumulation of GC-AuNPs was also demonstrated to be not involved in the initial tumor targeting (e.g., 1 h p.i.) and the very late retention stage (e.g., 72 h p.i.) of GC-AuNPs. The acidic tumor microenvironment temporarily enhanced the accumulation of ultrasmall GC-AuNPs in the acidic LNCaP tumors. In our group, by taking the advantages of controllable both pH-responsive imidazole ring functionalization and PEGylation, we developed a facile strategy to control the tumor-acidity activated charge-reversal behaviors and HDs of ultrasmall luminescent AuNPs (~ 1.7 nm) to achieve highly tumor-targeting specificity (Fig. 9d) [159]. Those ultrasmall AuNPs showed well–controlled HDs (2.4–4.2 nm) and ζ-potential values (− 31.2 to − 11.4 mV at pH 7.4), which were then investigated their tumor-targeting behaviors at both in vitro and in vivo levels. Under pH 7.4 in normal tissues, the AuNPs showed a highly negative charge (− 31.2 to − 11.4 mV), while in the acidic tumor microenvironment, the AuNPs transformed to positive charge (+ 1.4 to + 18.7 mV at pH 6.5). We discovered that the ultrasmall charge–reversal AuNPs with high ζ-potential values (− 11.4 mV at pH 7.4) and large HD (4.2 nm) contributed to the high tumor targeting efficiency (~ 9% ID g^−1^) with low nonspecific accumulation in MPS organs (e.g., liver ~ 2% ID g^−1^). It was also demonstrated that the optimized ultrasmall charge-reversal AuNPs (HD: 4.2 nm; ζ-potential: − 11.4 mV at pH 7.4) could rapidly (< 10 min) and selectively recognize small metastatic tumors (~ 1 mm) in liver and lung with high signal–to–noise ratios of 4.6 and 4.5, respectively.

## Imaging Performance

With the sharp size shrinking, the quantum confinement effect of the ultrasmall AuNPs leads to splitting electron energy levels, resulting in tunable fluorescent emissions with wavelengths from visible region to the NIR-II region (1000–1700 nm). The excellent intrinsic emissions and outstanding biological behaviors (e.g., renal clearance, low nonspecific accumulation and EPR effect) of the ultrasmall AuNPs show great potentials as unique optical probes to address many challenges in the healthcare field using fluorescence imaging. Strong multiple absorption bands in the visible to NIR region are observed from the ultrasmall AuNPs due to the single-electron transition [8, 207, 208], which enables the AuNPs with photoacoustic imaging capability. The ultrasmall AuNPs with high atomic number of Au (Z = 79) show excellent absorbers of X-rays and can offer excellent improvements in CT imaging [209, 210]. Furthermore, the surface of ultrasmall AuNPs can be functionalized to couple with other contrast agents (e.g., Gd^3+^, perfluorocarbon, ^198^Au and ^64^Cu) to generate multimodal imaging capabilities such as magnetic resonance imaging (MRI) [211], single-photon emission computed tomography (SPECT) [212] and positron emission tomography (PET) [213, 214].

### Fluorescence Imaging

**Fig. 10:**
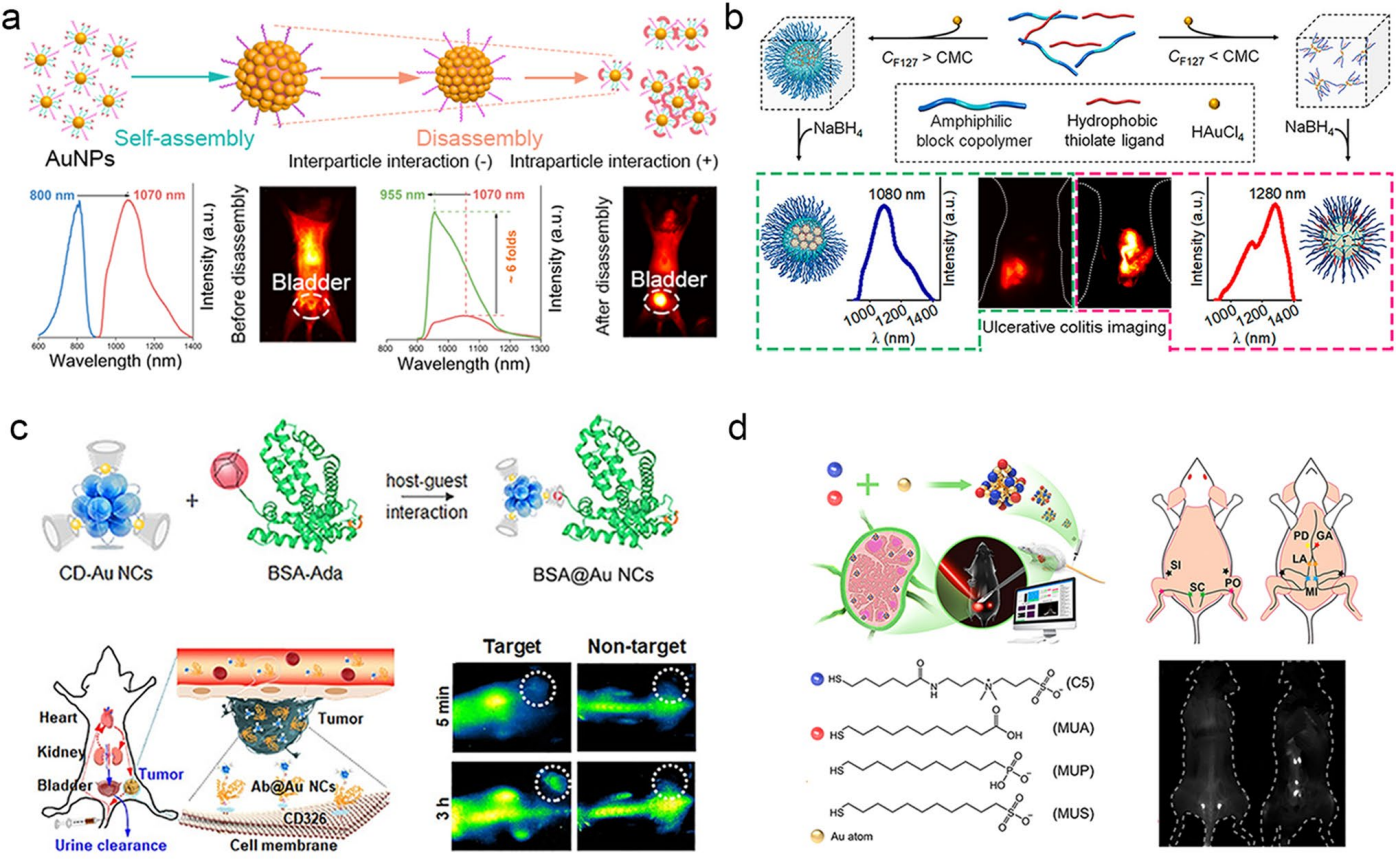
NIR–II fluorescence imaging of the ultrasmall AuNPs. **a** Highly controllable nanoassemblies of luminescent AuNPs with abnormal DIEE for in vivo imaging applications [221]. Copyright (2022), John Wiley and Sons. **b** Luminescent AuNPs with controllable hydrophobic interactions for colitis imaging [222]. Copyright (2022), American Chemical Society. **c** NIR–II AuNCs–based protein biolabels for in vivo tumor–targeted imaging [65]. Copyright (2020), John Wiley and Sons. **d** Multifunctional AuNCs for targeting, NIR–II imaging, and treatment of cancer lymphatic metastasis [225]. Copyright (2022), American Chemical Society

Fluorescence imaging in the NIR-II region shows great potentials in intravital biomedical imaging and analysis, which significantly overcomes the challenges of strong tissue absorption, auto-fluorescence and photon scattering to show deep tissue penetration (up to ~ 3 mm depth), micron-level spatial resolution, and high signal-to-background ratio [215–217]. With the emission redshifts to the NIR-II region from the visible region, the non–radiative transitions from the AuNPs become fast [218–220], resulting in low emission quantum yields (QYs) in the NIR-II region. It is a challenge to achieve water-soluble AuNPs with both high QYs and emission peaks > 1100 nm for biomedical observations. In our group, using the alterable siloxane bridge cross-linking states, we developed a facile strategy to fabricate water-soluble nanoassemblies of NIR-II AuNPs (1.2 nm) co-coated with both an organic silane and a hydrophilic thiolate polyethylene glycol [221], which showed significant disassembly-induced emission enhancement (DIEE) properties. The formed AuNP nanoassemblies with dominant interparticle crosslinking exhibited a maximum emission at 1070 nm with QYs of 1.8%. After disassembly, the AuNP nanoassemblies with increased intraparticle cross-linking showed a unique DIEE with the emission increased more than 6 folds to reach the QYs as high as 12%, which provided a facile pathway for designing highly-emissive AuNP nanoassemblies toward bioimaging (Fig. 10a). Furthermore, using an ABC template with controllable hydrophobic interactions in terms of unimers and micelles, we reported a facile strategy for red-shifting the emission and enhancing the biological interactions of luminescent AuNPs [222]. The highly red-shifted AuNPs with emission peak at 1,280 nm were generated with ABC unimers attached on the surface through strong intraparticle hydrophobic interactions for colitis imaging (Fig. 10b).

Recently, various ultrasmall AuNPs with emissions in the NIR-II region have been developed and their imaging performance have been investigated. Cheng et al. reported a Au_25_(SG)_18_ with an emission maximum at 1050 nm [223]. The synthesized Au_25_(SG)_18_ showed high capability binding to hydroxyapatite and accumulated in bone tissues. The in vivo NIR-II fluorescence imaging demonstrated that Au_25_(SG)_18_ showed a signal-background ratio (SBR) as high as 4.35 at 24 h p.i. in the identification of spine from the surrounding soft tissue. Zhang et al. reported an Au_25_NCs (1.8 nm) with emission center of 1120 nm [224]. The dynamic NIR–II fluorescence imaging was then used to analyze blood perfusion of arterial vessel in brain. The observed blood perfusion rate in the injured left brain (0.11 s^−1^) was two times lower than the normal right brain (0.24 s ^−1^). Yang et al. synthesized a cyclodextrin (CD) -protected AuNCs (CD-AuNCs, ~ 1.85 nm) with NIR-II emission at ~ 1050 nm for protein/antibody labeling through host-guest chemistry [65]. The CD-AuNCs-labelled anti-CD326 antibody (Ab@Au NCs) exhibited a threefold increase in NIR-II signal intensity as compared to the CD-AuNCs without labelling (Fig. 10c). Jiang et al. reported a series of ligand-/multiligand-capped AuNCs (1.2 nm) with emissions at 1000–1100 nm [225]. These AuNCs were then utilized for fluorescence imaging of lymph-node (LN) cancer metastasis, and the AuNCs with optimized surface chemistry showed a SBR of approximately 60 in the LN region with a long imaging window (> 3 h) (Fig. 10d). Xiao et al. developed a AuNCs (1.6 nm) with emission at 1050 nm for pH monitoring in stomach [226]. Methylene blue was loaded on the surface of polydopamine-encapsulated AuNCs to quench the emission of AuNCs through photo-induced electron transfer. Under the stimulation of gastric acid, the protonation of the cationic polymer caused the detachment of methylene blue to recover the emission of AuNCs for gastric acid imaging. Dai et al. synthesized a GS-AuNCs (~ 1.6 nm) with maximum emission at 1090 nm [227]. After phosphorylcholine functionalization, the AuNCs showed minimal binding to serum proteins and efficient renal clearance (93% ID, 24 h p.i.), which were then used for imaging the draining LNs in 4T1 mouse breast cancer and CT26 mouse colon cancer, respectively. The NIR-II imaging showed a SBR as high as 22 when the 1300 nm long–pass emission filter was used.

### CT Imaging

**Fig. 11:**
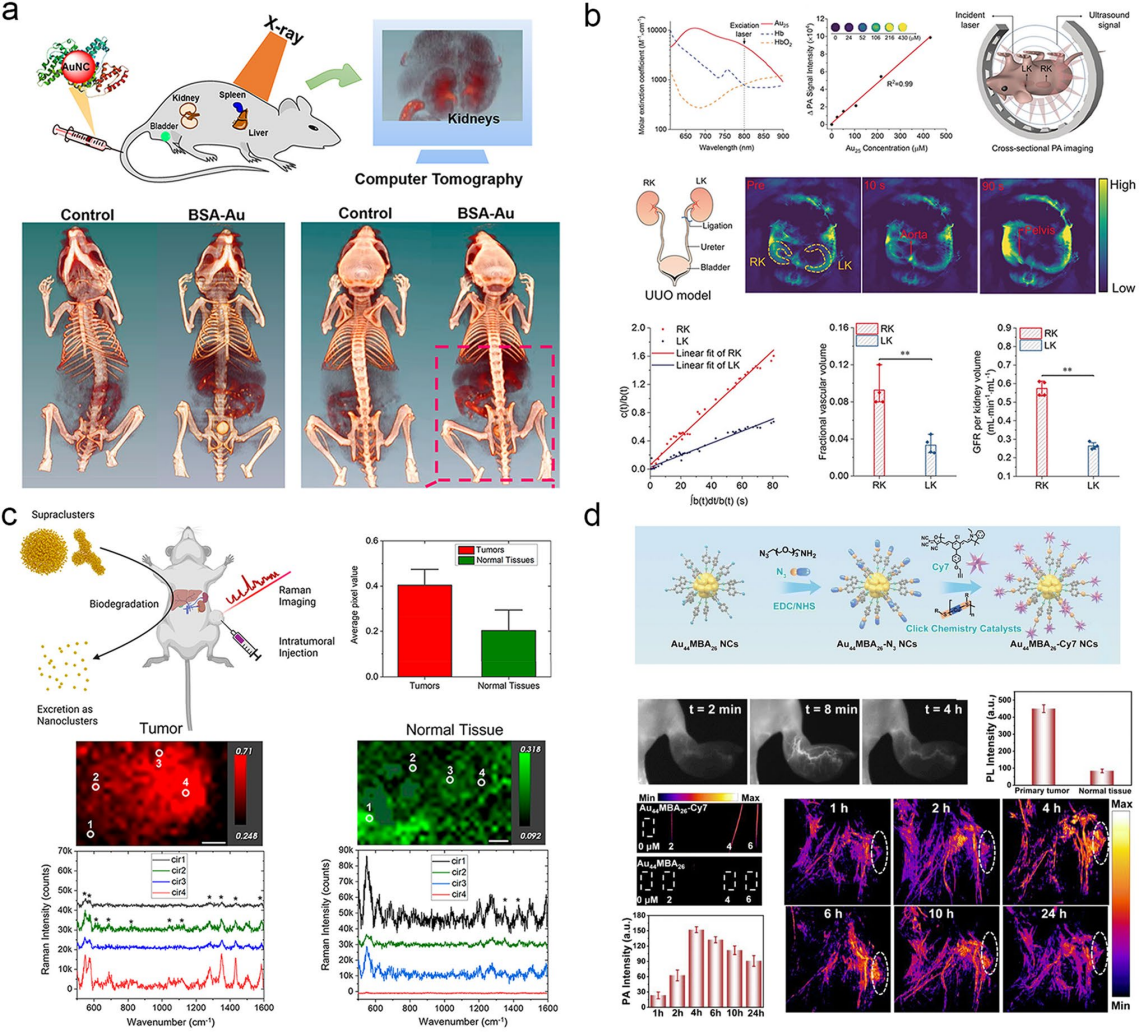
Multifunctional imaging of ultrasmall AuNPs. **a** The AuNCs used for in vivo 2D and 3D CT of murine kidneys [68]. Copyright (2015), American Chemical Society. **b** Photoacoustic imaging of AuNPs transport in the kidneys [228]. Copyright (2019), John Wiley and Sons. **c** Gold supraclusters for in vivo Raman imaging of tumors [67]. Copyright (2023), American Chemical Society. **d** In vivo NIR-II fluorescence imaging and photoacoustic imaging of Au_44_NCs [66].Copyright (2023), Royal Society of Chemistry

Ultrasmall AuNPs with high atomic numbers (Z = 79) with strong absorption of X–rays can serve as CT contrast agents to image internal organs such as kidneys, where the larger ones cannot reach. Gao et al. reported an albumin-stabilized AuNCs (1.4 nm), which exhibited red emission at 645 nm and stable X-ray attenuation [68]. The synthesized AuNCs showed the slope of HU (Hounsfield Units) values (17.85), which was 4.3 times higher than that of the clinical CT contrast agent iopromide (4.15) (Fig. 11a). Zheng et.al reported that the GS-AuNPs (2 nm) could be used for real-time accumulation observation in the bladder area using CT imaging [155]. The CT intensity of GS–AuNPs at a concentration of 9 mg mL^−1^ exhibited a high slope of HU values (845 HU). Basilion et al. used prostate specific membrane antigen targeting ligand (PSMA-1) as surface ligand to synthesize ultrasmall Au_25_ for prostate cancer targeting CT imaging and radiotherapy enhancement [157]. By the advantage of targeting type II membrane proteins highly expressed in most prostate cancers, the CT value of the PC3pip tumor (PSMA-positive) was 374 HU, twice as high as that of the PC3flu tumor (PSMA-negative) site (195 HU, at 4 h p.i.).

### Multimodal Imaging

The ultrasmall AuNPs with strong absorption in the visible and NIR region can serve as contrast agents for photoacoustic imaging [229]. Using the strong NIR absorption of Au_25_(SG)_18_, Zheng et.al reported that Au_25_(SG)_18_ could be used for photoacoustic imaging in the visualization of the in situ transportation from the aorta to the renal parenchyma with a temporal resolution of 1 s [228]. This high temporal and spatial resolution photoacoustic imaging were then used for precise quantification of glomerular filtration rate in the normal (0.26 ± 0.02 mL min^−1^ mL^−1^) and diseased kidneys (0.57 ± 0.04 mL min^−1^ mL^−1^). In addition, the facile functionalization of ultrasmall AuNPs allows the construction of multi-modal imaging probes, which integrates the advantages of different imaging techniques to obtain important information from complicated organisms (Fig. 11b). Zheng et al. reported a one–step synthesis of 810 nm-emitting radioactive AuNPs incorporated with a ^198^Au radioisotope (GS–[^198^Au]AuNPs, 2.6 nm) [156], which were then used for both SPECT imaging and fluorescence imaging of the pharmacokinetics. The GS–[^198^Au]AuNPs were renal clearable and exhibited molecular pharmacokinetics with a rapid *t*_1/2α_ of 5.0 min and a *t*_1/2β_ of 12.7 h. Wang et al. developed a 810 nm-emitting AuNCs encapsulated with a fluorinated polymer (AuNCs@PF) [230], which were capable of fluorescence imaging, fluorine MRI and CT imaging. Jin et al. constructed pH-responsive Raman-active renal-clearable metallic superclusters (> 50 nm) from the assembly of AuNCs (2–3 nm) [67]. The glutathione diethyl ester-capped AuNCs was synthesized and loaded with a NIR-resonant Raman dyes (BS2G) to create the pH-responsive superclusters capable of in vivo Raman imaging in acidic tumor environments. The average pixel intensity in the 4T1 tumor (0.4 ± 0.2) was 2.2 times higher than that of the normal tissue (0.2 ± 0.04) (Fig. 11c). Yuan et al. designed NIR–II luminescent Au_44_NCs (1.6 nm) with emissions at both 1080 and 1280 nm by conjugating an aromatic photoacoustic/photothermal molecules (Cy7) through a click chemistry [66], which were then used for both NIR–II fluorescent and photoacoustic imaging–guided photothermal therapy (Fig. 11d).

## Conclusion and Perspective

The fundamental understanding on the nano–bio interactions of ultrasmall AuNPs is an important issue in the development of translatable intelligent nanomedicine with both maximum efficacy and minimum toxicity toward disease theranostics. In this review, we briefly summarize the recent advances of biological interactions and imaging of ultrasmall AuNPs at both in vitro and in vivo levels including the cellular interactions, organ interactions, tumor interactions and imaging performance. The fundamental physicochemical properties of ultrasmall AuNPs, such as surface charge, surface coverage, hydrophobicity, functionality and concentration, play important roles in governing their nano–bio interactions with cells, organs and tumors. By taking the advantages of unique quantum confinement effect, high atomic number of Au and easy functionality, the ultrasmall AuNPs with the excellent intrinsic emissions and outstanding biological behaviors show great potentials as promising multimodal probes to address many challenges in the healthcare field using the imaging techniques such as fluorescence imaging, CT imaging, photoacoustic imaging, MRI imaging, PET imaging and Raman imaging.

With the above advances, the future biomedical applications of ultrasmall AuNPs hold great promise. However, the research on the nano–bio interactions of ultrasmall AuNPs is still in their early stage. An in-depth understanding of the nano–bio interactions of ultrasmall AuNPs highly relies on the development of synthetic strategies towards water-soluble different-sized atomically precise AuNCs to overcome the long-standing size heterogeneity issue of the nanoparticles, so that the precisely understanding of size-dependent nano–bio interactions of ultrasmall AuNPs will be achieved. In addition, the understanding on whether and how ultrasmall AuNPs cross the biological barriers such as blood brain barrier (BBB) [231, 232], the small intestine and peripheral nerves [233], should be further investigated, which will facilitate a better understanding of their in vivo toxicity and also have a significant clinical impact. Furthermore, in order to achieve more accurate and multidimensional dynamic information of the nano–bio interactions, researchers are highly suggested to pay more attention to the theoretical simulation investigations of the ultrasmall AuNPs in the biological systems. In summary, nano–bio interaction dictates the overall biological applications of the ultrasmall AuNPs. A systematic understanding the complicate nano–bio interactions thus provide useful insights into the nanotoxicity, in vivo transport, targeting, excretion and other key properties (e.g., inflammation and immunity) of these novel ultrasmall nanoparticles to facilitate the future clinical translation.
